# Supplementary material for: A high-resolution mRNA expression time course of embryonic development in zebrafish
Source: eLife. 2017 Nov 16;6:e30860. doi: 10.7554/eLife.30860 (PMC5690287; doi:10.7554/eLife.30860)
Supplement: Supplementary file 6. [file elife-30860-supp6.zip › biolayout-clusters-files/Cluster214-genes.html]

Cluster214


# Cluster214: Genes

| Ensembl ID | Gene Name | Chr | Start | End | Biotype |
| --- | --- | --- | --- | --- | --- |
| ENSDARG00000101160 | apoa4a | 19 | 10927028 | 10931909 | protein\_coding |
| ENSDARG00000016706 | atic | 1 | 4739585 | 4806325 | protein\_coding |
| ENSDARG00000035602 | dao.1 | 5 | 19684638 | 19691550 | protein\_coding |
| ENSDARG00000103759 | fahd2a | 8 | 41152055 | 41194562 | protein\_coding |
| ENSDARG00000027529 | hmox1a | 3 | 25880611 | 25886721 | protein\_coding |
| ENSDARG00000023157 | si:rp71-1g18.7 | 2 | 37971243 | 37974440 | protein\_coding |
| ENSDARG00000102241 | zgc:136410 | 21 | 44510841 | 44518121 | protein\_coding |
